# Predicting Depression From Smartphone Behavioral Markers Using Machine Learning Methods, Hyperparameter Optimization, and Feature Importance Analysis: Exploratory Study

**DOI:** 10.2196/26540

**Published:** 2021-07-12

**Authors:** Kennedy Opoku Asare, Yannik Terhorst, Julio Vega, Ella Peltonen, Eemil Lagerspetz, Denzil Ferreira

**Affiliations:** 1 Center for Ubiquitous Computing University of Oulu Oulu Finland; 2 Department of Clinical Psychology and Psychotherapy Ulm University Ulm Germany; 3 Department of Medicine University of Pittsburgh Pittsburgh, PA United States; 4 Department of Computer Science University of Helsinki Helsinki Finland

**Keywords:** mHealth, mental health, mobile phone, digital biomarkers, digital phenotyping, smartphone, supervised machine learning, depression

## Abstract

**Background:**

Depression is a prevalent mental health challenge. Current depression assessment methods using self-reported and clinician-administered questionnaires have limitations. Instrumenting smartphones to passively and continuously collect moment-by-moment data sets to quantify human behaviors has the potential to augment current depression assessment methods for early diagnosis, scalable, and longitudinal monitoring of depression.

**Objective:**

The objective of this study was to investigate the feasibility of predicting depression with human behaviors quantified from smartphone data sets, and to identify behaviors that can influence depression.

**Methods:**

Smartphone data sets and self-reported 8-item Patient Health Questionnaire (PHQ-8) depression assessments were collected from 629 participants in an exploratory longitudinal study over an average of 22.1 days (SD 17.90; range 8-86). We quantified 22 regularity, entropy, and SD behavioral markers from the smartphone data. We explored the relationship between the behavioral features and depression using correlation and bivariate linear mixed models (LMMs). We leveraged 5 supervised machine learning (ML) algorithms with hyperparameter optimization, nested cross-validation, and imbalanced data handling to predict depression. Finally, with the permutation importance method, we identified influential behavioral markers in predicting depression.

**Results:**

Of the 629 participants from at least 56 countries, 69 (10.97%) were females, 546 (86.8%) were males, and 14 (2.2%) were nonbinary. Participants’ age distribution is as follows: 73/629 (11.6%) were aged between 18 and 24, 204/629 (32.4%) were aged between 25 and 34, 156/629 (24.8%) were aged between 35 and 44, 166/629 (26.4%) were aged between 45 and 64, and 30/629 (4.8%) were aged 65 years and over. Of the 1374 PHQ-8 assessments, 1143 (83.19%) responses were nondepressed scores (PHQ-8 score <10), while 231 (16.81%) were depressed scores (PHQ-8 score ≥10), as identified based on PHQ-8 cut-off. A significant positive Pearson correlation was found between screen status–normalized entropy and depression (*r*=0.14, *P*<.001). LMM demonstrates an intraclass correlation of 0.7584 and a significant positive association between screen status–normalized entropy and depression (β=.48, *P*=.03). The best ML algorithms achieved the following metrics: precision, 85.55%-92.51%; recall, 92.19%-95.56%; F1, 88.73%-94.00%; area under the curve receiver operating characteristic, 94.69%-99.06%; Cohen κ, 86.61%-92.90%; and accuracy, 96.44%-98.14%. Including age group and gender as predictors improved the ML performances. Screen and internet connectivity features were the most influential in predicting depression.

**Conclusions:**

Our findings demonstrate that behavioral markers indicative of depression can be unobtrusively identified from smartphone sensors’ data. Traditional assessment of depression can be augmented with behavioral markers from smartphones for depression diagnosis and monitoring.

## Introduction

### Background

Depression is one of the most prevalent, complex, and heterogeneous mental health challenges of our time. In 2020, the World Health Organization (WHO) estimated that depression has impacted 264 million people worldwide [1], and it is projected to be the leading contributing factor to global disease burden by 2030 [2]. In these individuals, depression inflicts recurrent episodes of guilt, sadness, cognitive impairments, suicidal ideation, and sleep disturbances [1,3-5]. Depression increases the risk and medical costs of many medical disorders such as stroke, Parkinson, or Alzheimer [6-11]. Depression is treatable with psychotherapy and medication. Yet, in many individuals with depression, it remains undiagnosed and untreated due to barriers such as social stigma and inaccurate assessment methods [1,3,12,13]. The ability to detect early warning signs of depression, continuously and as effortlessly as possible, by extending current assessment methods could have a significant impact in mitigating or addressing depression and its related negative consequences [3,10,11,14].

For the past 30 years, clinician-administered and self-reported questionnaires remain the gold standard in the assessment and diagnosis of depression [3,13]. However, the limitations of these traditional depression assessment methods have been debated. Such methods are applied sparingly (eg, a couple of times within a year), thus missing out on the moment-by-moment behavioral patterns of individuals between health assessments. Lastly, self-reported appraisals are affected by memory and recall biases in reconstructing past events and may be prone to socially desirable reporting from individuals [12,15-17].

Today, smartphones and wearables offer a unique opportunity to overcome limitations in traditional depression assessment methods. Smartphones and wearables (eg, Fitbit, Oura Rings, and smartwatches) have become ubiquitous in the global population, they are inherently personal, and people are continuously monitored through their embedded sensors (eg, camera, accelerometer, global positioning system [GPS], Bluetooth, and many more) [18,19]. Instrumenting smartphones and wearables to capture in situ, fine-grained, and moment-by-moment data sets with sensing apps [20-24] has made it possible to passively collect data sets in naturalistic settings. Inherent in these data sets are behavioral patterns: routines, rhythms, activities, and interactions that are useful in complementing traditional depression assessment methods, in studying the mental health of individuals, and in developing timely mental health interventions [14,22,25-28].

### Related Work

A growing body of research in smartphone and wearable sensing, human behavior modeling has improved our understanding of the relationship between mental health and biomarkers [3,20-22,26,27,29-31]. In medicine, biomarkers are pathological, anatomical, or physiological characteristics that are quantified and evaluated as indicators of a biological process or a response to medical interventions [23]. Here, we define biomarkers (or digital biomarkers) of mental health as quantifiable behaviors (or features) extracted from smartphone or wearable data that can be monitored and collected over time to objectively assess mental health and effectiveness of interventions. Monitoring these biomarkers’ fluctuations is essential in the early detection and treatment of mental health disorders [3,32]. For example, in Alzheimer disease, biomarkers such as cognitive, sensory, and motor degeneration precedes clinical diagnosis for about 10 or 15 years [32].

In the StudentLife study [22], for example, geolocation, sleep, and activity-based biomarkers were extracted from a data set collected from 48 students over 10 weeks. Significant correlations were found between the following digital biomarkers: sleep duration (*r*=–0.360), activity duration (*r*=–0.388), traveled distance (*r*=0.338), and various mental health symptoms. Similarly, Wang et al [3] collected data sets with the StudentLife sensing app from 83 college students across two 9-week terms. Significant correlations were found between depression and accelerometer-based biomarkers (mean stationary time, *r*=0.256) and screen usage–based biomarkers (mean unlock duration, *r*=0.283). With an analysis of variance, a significant difference in unlock duration (*F*=5.733) was found between depressed and nondepressed groups. Saeb et al [33] in their study of 40 participants for 2 weeks also found a statistically significant correlation between depression and GPS location–based biomarkers (ie, variance in locations visited, [*r*=0.58] and regularity in 24-hour movement rhythm [*r*=0.63]) and phone usage–based biomarkers (ie, phone usage frequency [*r*=0.54]).

Another promising source of biomarkers is wearable devices [30].

Actigraphy-based biomarkers that quantify time sequences of rest and active behaviors with accelerometer sensors are known to be useful in predicting mood disorders such as depression and bipolar disorder [34,35]. In a 2-week study on 23 participants, Jacobson et al [34] extracted biomarkers from data sets collected with wrist-worn actigraph watches. A machine learning (ML) model trained with these digital biomarkers could predict the depression status of participants with high accuracy (ie, accuracy 89%, Cohen κ=0.773). In another 2-week study [35] on 40 geriatric participants, accelerometer-based digital biomarkers were extracted from wrist-worn actigraph watches. With 4 ML models, the study found that these biomarkers could predict depression with a high accuracy (ie, accuracy 0.910, precision 0.929, and specificity 0.940). Other promising biomarkers from wearable devices are heart rate variability, which has been found to be consistently lower in patients with psychiatric disorders, electrodermal activity, and skin conductance [36,37].

Taken together, previous research has shown the potential of quantifying human behavior from smartphones and wearables data set as biomarkers. These biomarkers are insightful in understanding depression.

### Objectives

In this study, we aim to investigate the feasibility of predicting depression using digital biomarkers quantified from a smartphone data set. To this end, we explore the relationship between digital biomarkers and depression severity with statistical methods. We investigate whether depression can be predicted with digital biomarkers using supervised ML algorithms.

## Methods

### The Data Set

We utilized an existing data set collected in a longitudinal observational study with the Carat app [21], derived from a cohort of anonymous Android participants worldwide [38]. The data set was collected from 843 participants between March and August 2018 (~6 months).

The Carat app is a mobile sensing app, originally developed by a team of researchers from the University of Helsinki and the University of California, Berkeley, for smartphone energy consumption research [21,39]. The Carat app is freely available on mobile app stores and gives users personalized smartphone battery consumption reports. Anonymous users worldwide who install the Carat app may voluntarily be recruited to contribute their data set to research.

The data set used in this study was a subset of the large-scale crowdsourced Carat app data set from anonymous volunteers. The study data set was collected for a multifaceted purpose, which includes studying the relationship between smartphone app usage and Big 5 personality traits [40]; studying the similarities and differences in demographic, geographic, and cultural factors of smartphone usage [38]; and mental health research. The advertisement for the recruitment of participants was sent as push notifications through the Carat app to 25,323 verified users (ie, users with matching time zone and mobile country code) [38].

All participants in this data set are Android-based smartphone users, who explicitly and voluntarily gave their consent from their mobile devices after they were informed about the purpose of the data collection, the data collection procedures, and management of the data set. The data set does not contain personally identifiable information, and was collected under the institutional review board license from the University of California, Berkeley and the University of Helsinki [21,40].

### Mobile Sensing Variables Collected by Carat

Besides battery consumption data, the Carat Android app unobtrusively collected participants’ time zone and time-stamped data, including foreground app usage (ie, app the participant has interacted with), internet connectivity (ie, connected and disconnected states), and screen lock and unlock logs. This data set was sampled at each 1% battery change (ie, while charging or discharging) on the participants’ smartphone. The Carat app also collected participant’s demographic information, including age, gender, education, and occupation via a self-report.

### Mental Health Assessment

In addition to the mobile sensing and demographic variables, depression severity was assessed by a self-report instrument. Participants answered the 8-item Patient Health Questionnaire (PHQ-8) [41] at 2-week intervals as specified by the PHQ-8 protocol. Although the depression assessments were self-reported, the PHQ-8 is clinically validated for the assessment of depression severity, has high internal consistency (Cronbach α=.82), and has been used in several previous studies [3,7,41]. PHQ-8 measures depression severity for the past 2 weeks with items such as “Little interest or pleasure in doing things,” “Feeling down, depressed, or hopeless,” “Trouble falling or staying asleep, or sleeping too much.” Each item of the PHQ-8 is scored on a scale from 0 (Not at all) to 3 (Nearly every day). The total PHQ-8 score ranges from 0 to 24, with a score of 10 or more indicating major depression or other severe forms of depression [41].

### Data Inclusion and Exclusion

For each participant’s data set, we excluded days with at least 10 missing log intervals (ie, days where no data were logged by the Carat app for at least 10% battery charging or discharging periods). Next, we only included PHQ-8 responses from participants with at least 8 days of data within the preceding 2 weeks of PHQ-8 response. Consequently, the final data contained 629 participants, 1374 PHQ-8 responses with 13,898 days of participants’ data set.

### Feature Engineering

#### Characterization of Data Set

Our data set is primarily categorized into screen status, internet connectivity, and foreground app usage logs. For data preprocessing, we converted the time stamps of the data set to local date and time, using the participants’ time zone. We computed digital biomarkers (herein features) by quantifying the per-participant hourly and daily behavioral patterns (ie, routines, irregularity, variability) from these data sets with simple counts, SDs, entropy [6,14,25,42-45], and regularity index [27,44] measures. All computed features were merged per participant at the day level.

#### Entropy

We computed entropy to capture the degree of variability, complexity, disorder, and randomness in the participant behavior states from screen status (ie, on and off states), internet connectivity (ie, disconnected and connected states), and foreground app (ie, the frequency of use per app) over a 24-hour period of each day. Entropy was calculated using the Shannon entropy [45] formula:


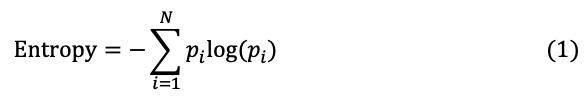


where *N* is the number of states and *p_i_* is the percentage of the state *i* in the time series data. For example, a higher screen status entropy reflects the fact that the participant’s screen on and off pattern is more distributed between on and off states, albeit with a high degree of uncertainty and complexity in the transition between the screen on and off states in a 24-hour period. Conversely, a lower screen status entropy reflects that fact that the participant’s screen is much often in one state (on or off) over a 24-hour period. In addition to entropy, we computed normalized entropy as the entropy divided by log(*N*).

#### Regularity Index

Regularity index quantifies routines in participant behaviors by capturing the similarity (or difference) in participant behaviors between the same hours across different days. For internet connectivity, for instance, the regularity index quantifies the routineness of the participant’s internet connectivity behavior at the same hours (eg, every 9 am) for all days. We determined the hourly values as follows: for screen status, the modal screen status for each hour; for internet connectivity, the modal connectivity state for each hour; and for foreground app usage, the number of distinct apps usage for each hour.

Following the regularity index computation method of Wang et al [44], we computed the regularity index of the screen, internet connectivity, and foreground app usage for days *a* and *b* using the formula


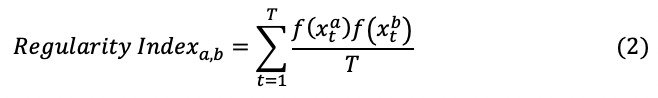


where *a* and *b* are 2-day pairs, *T*=24 hours, and 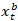 is the rescaled (ie, between –0.5 and 0.5) value of hour *t* of day *b*. For each day, we computed the average regularity index for all combinations of that day and other days of the week.

#### Standard Deviation and Counts

The SD features capture the variance of daily behavior between 4-day epochs based on the hour of the day. We defined morning as the 6-11th hour, afternoon as the 12-17th hour, evening as the 18-23rd hour, and night as the 0-5th hour of the day. We computed the count of each screen status, the count of each internet connectivity status, and the count of foreground app usage per day epoch. With these counts per day epoch, we computed the SD per day.

We also computed the day level count of each screen status, the count of each internet connectivity status, and the count of foreground app usage. Additionally, we computed the count of minutes until the first and last use of foreground app per day.

### Correlation and Association Analysis

Before beginning the statistical analysis, we pooled (ie, aggregated) the extracted features within the preceding 2 weeks (ie, assessment window) of each PHQ-8 response from a participant. The pooling is to ensure that the timelines of the feature variables in the analysis are aligned with those of the PHQ-8 assessment window. The pooling was done as follows: for each PHQ-8 response, we pooled all entropy and regularity index features by computing the average feature values for all days within the PHQ-8 assessment window. Instead of average values for SD, we took a different approach due to the additive properties of SD measures. For SD features, we computed the pooled SD [46,47].

For correlation analysis, we used the pooled data to quantify the linear relationship between the features and depression severity (ie, PHQ-8 responses). The correlations were computed using the Pearson correlation coefficient. Full information maximum likelihood [48-50] was used in the correlation analysis to avoid biases introduced by missing data. We used the Holm–Bonferroni [51] method to adjust the *P* values for multiple testing, with a false discovery rate of 0.05.

For association analysis, we used the bivariate linear mixed model (LMM) [8,44,52-55] to study the association between the pooled features and the PHQ-8 response. The data set in this study is a longitudinal data set with repeated measures from the same individuals. Given this nested structure, the assumption of normally and independently distributed residuals would be violated in linear regression models. Hence, we opted for LMM, which takes into account fixed and random variations in the data set in respect of a grouping variable, the participant in this case. LMM also reduces the likelihood of Type I error [56]. To verify our decision for LMM, we computed the intraclass correlation (ICC). ICC > 0.05 necessitates LMM.

In the LMM, we used multiple imputation to handle missing data, taking into account the nested structure of the data set [57]. Using predictive mean matching for multilevel data [58], a total of 20 imputed data sets were generated. We used Robin’s rule [59] to pool the results of the LMM run with each imputed data set. In the LMM analysis, all features were normalized to have a 0 mean and a unit SD. To address multiple comparison problems, we adjusted the *P* values in the LMM to control the false discovery rate with the Benjamini–Hochberg procedure [60,61]. Adjusted *P* value <.05 was considered to be significant.

### Predictive Analysis With Machine Learning

#### Machine Learning Setup

We developed population-based supervised ML classifiers to explore how the digital biomarkers/features predict the depression state of an individual. We also explored whether including participants’ self-reported demographics as features would improve the ML classifier performance.

To this end, we used 5 supervised ML models: random forest (RF), support vector machine (SVM) with radial basis function (RBF) kernel, XGBoost (XGB) [62], K-nearest neighbor (KNN), and logistic regression (LR). With the RBF kernel, an SVM classifier, a linear classifier, can classify nonlinear data sets [63,64]. These algorithms have been used in previous work [65-69] on mental health studies. We used the same pooled and imputed data set from statistical analysis, but ensured that all records are distinct. We labeled our data set with 2 classes, based on PHQ-8 scoring guidelines, where PHQ-8 score ≥ 10 is depressed (label 1) and PHQ-8 score <10 is nondepressed (label 0). We created 2 data sets for the ML modeling: (1) a data set with PHQ-8 scores as labels, and digital biomarkers/features as predictors, and (2) a second data set with PHQ-8 scores as labels and digital biomarkers/features, age group, and gender as predictors. The age group and gender demographics were converted from categorical to numerical data using one-hot encoding [63,70].

All the ML modeling was performed using stratified and nested cross-validation [71,72]. The nested cross-validation is a state-of-the-art procedure to prevent overfitting and overestimation of the hyperparameters of the ML classifiers. Stratified 10 folds in the outer cross-validation and stratified 3 folds in the inner cross-validation were used. With this approach, for each iteration of the outer cross-validation, 1 stratified fold was used as a testing data set. The remaining 9 stratified folds were used for hyperparameter optimization in the inner cross-validation.

The hyperparameter optimization in the inner cross-validation was done with grid search over a grid of parameters, where all combinations of hyperparameters are exhaustively considered. We use the F1 (macro averaged) score to select the most optimized hyperparameters.

#### Imbalanced Data Handling

It is worth noting that in the ML setup, we used stratified sampling in the nested cross-validation. The stratified sampling ensures the splitting of the data set into folds that have an equal proportion of each class (ie, labels 1 and 0). However, the proportion of each class is still dependent on its availability in the data set. We handled class imbalance in the training data set with the synthetic minority over-sampling technique (SMOTE) [65,73,74], which generates synthetic data for the minority class, resulting in a balanced training data set.

#### Feature Analysis

We used the permutation importance method [75,76] to compute the importance of features. The permutation importance method is model agnostic and computes the proportional decrease in a model score when features are randomly shuffled. We used the area under the curve (AUC) receiver operating characteristic as the model in the permutation importance computation. We computed the feature importance using the test set of the outer cross-validation. For each ML classifier, we ranked the features by the average feature importance computed across all 10 folds of the outer cross-validation.

#### Model Evaluation

We created 2 baseline classifiers to benchmark the performance of the ML classifiers. The first baseline is a random weighted classifier (RWC) with 10,000 randomly generated predictions based on a multinomial distribution of the nondepressed and depressed classes. The second baseline is a decision tree (DT) classifier trained using the same approach as the ML classifiers, but with age group and gender as features. The performance of the ML classifiers and baseline classifiers was measured using the following performance metrics: accuracy, precision, recall, AUC, F1 score, and Cohen κ. The precision, recall, and F1 scores were computed with an emphasis on predicting the depressed score (ie, label 1).

### Software

Data preprocessing and feature extraction pipeline were created with Python (version 3.7.6) and R (version 4.0.2) programming languages, using Snakemake [77] and RAPIDS [78] for workflow management. All statistical analysis was performed in R, with mice [79] package for multiple imputation, lmer4 [80] and lmerTest [81] packages for LMM, and psych [50] package for computing correlation. All the ML was done in Python, with scikit-learn [63], imbalanced-learn [33], and XGB library [62,82].

## Results

### Participants’ Demographics

Self-reported demographic data from the 629 participants included in our analyses show that 69/629 (10.97%) were females, 546/629 (86.8%) were males, and 14/629 (2.2%) were nonbinary or preferred not to disclose their gender.

For the participants’ age distribution, 73/629 (11.6%) were aged between 18 and 24, 204/629 (32.4%) were aged between 25 and 34, 156/629 (24.8%) were aged between 35 and 44, 166/629 (26.4%) were aged between 45 and 64, and 30/629 (4.8%) were aged 65 years and over. The participants were distributed across at least 56 different countries, including 91/629 (14.5%) from unknown countries, 199/629 (31.6%) from the USA, 66/629 (10.5%) from Finland, 32/629 (5.1%) from Great Britain, 42/629 (6.7%) from Germany, and 29/629 (4.6%) from India. The data set also has participants from varied educational and occupational backgrounds. Table 1 provides summary statistics of the 629 participants in this study.

**Table 1:** Summary statistics of participants who were included in the data analysis (N=629).

Variable			Value, n (%)
**Age (years)**			
	18-24	73 (11.6)	
	25-34	204 (32.4)	
	35-44	156 (24.8)	
	45-64	166 (26.4)	
	≥65	30 (4.8)	
**Gender**			
	Female	69 (11.0)	
	Male	546 (86.8)	
	Other or Rather not tell	14 (2.2)	
**Education**			
	Elementary school/basic education	9 (1.4)	
	High school/sixth form/other upper secondary level	98 (15.6)	
	No education or rather not to tell	5 (0.8)	
	Professional graduate degree/higher university degree (master’s or equivalent)	193 (30.7)	
	Research graduate degree (PhD or equivalent)	34 (5.4)	
	Undergraduate degree/lower university degree (bachelor’s or equivalent)	228 (36.2)	
	Vocational school/trade school/other education leading to a profession	62 (9.9)	
**Occupation**			
	Agricultural forestry or fishery	1 (0.2)	
	Clerical support	14 (2.2)	
	Craft and trade or plant and machine operations	8 (1.3)	
	Entrepreneur or freelancer	30 (4.8)	
	Manager	59 (9.4)	
	No suitable option or rather not to tell	34 (5.4)	
	Professional	227 (36.1)	
	Retired	39 (6.2)	
	Sales or services	29 (4.6)	
	Staying at home (eg, with kids)	5 (0.8)	
	Student	74 (11.8)	
	Technician or associate professional	90 (14.3)	
	Unemployed or between jobs	19 (3.0)	
**Country**			
	Unknown	91 (14.5)	
	USA	199 (31.6)	
	Finland	66 (10.5)	
	Great Britain	32 (5.1)	
	Germany	42 (6.7)	
	Canada	16 (2.5)	
	India	29 (4.6)	
	Other^a^	154 (24.5)	

^a^Comprising 49 different countries with less than 15 participants, including South Africa, Morocco, Brazil, Philippines, Qatar, Japan, Russia, and Denmark.

### Smartphone Data and PHQ-8 Distribution

We had 1374 PHQ-8 responses. Table 2 presents the distribution of participants and their corresponding number of responses in the PHQ-8 data set. All PHQ-8 responses were collected every 2 weeks. At the minimum, 316/629 (50.2%) participants responded 1 time to the PHQ-8 depression assessment, and at the maximum, 1/629 (0.2%) participants responded 7 times. The mean number of responses per participant is 2.18 (SD 1.57).

For the distribution of the PHQ-8 scores, 1143/1374 (83.19%) responses were nondepressed scores (PHQ-8 score <10), while 231/1374 (16.81%) were depressed scores (PHQ-8 score ≥10). The mean PHQ-8 score is 5.19 (SD 5.22; range 0-24).

The number of smartphone data set days was 13,898 for all 629 participants. Table 3 shows the distribution of participants and their corresponding number of days in the smartphone data set. The mean number of days per participant is 22.1 (SD 17.90; range 8-86).

**Table 2:** Distribution of participants’ contribution to the PHQ-8^a^ responses (N=629).

Participants, n (%)	PHQ-8 responses, n
316 (50.2)	1
129 (20.5)	2
57 (9.1)	3
47 (7.5)	4
40 (6.4)	5
39 (6.2)	6
1 (0.2)	7

^a^PHQ-8: 8-Item Patient Health Questionnaire.

**Table 3:** Distribution of participants’ smartphone data set days (N=629).

Days, n	Participants, n (%)
8-14	364 (57.9)
15-28	126 (20.0)
29-42	53 (8.4)
43-56	34 (5.4)
57-70	29 (4.6)
71-84	22 (3.5)
85-98	1 (0.2)

### Features Engineered From Smartphone Data Set

In all, we computed 22 features from the smartphone data set. All features were aggregated at the day level. For example, the *screen_offCount* feature is the count of all screen off states during the day. We summarize the engineered features in Multimedia Appendix 1.

### Correlation and Association Between Features and Depression

We found a significant positive correlation between screen status–normalized entropy and depression (*r*=0.14, *P*<.001). We found no significant correlation between other screen, app, and internet connectivity features and PHQ-8 depression score. Multimedia Appendix 2 presents the full Pearson correlation coefficients and adjusted *P* values, with the Holm–Bonferroni method, between features and PHQ-8 depression score.

Regarding the association analysis, we found an ICC of 0.7584; thus, 75.84% of the variations in the features are explainable by the interindividual differences. We found a significant positive association between screen status–normalized entropy and depression (β=.48, *P*=.03). We found no significant association between other screen, app, and internet connectivity features and depression. Multimedia Appendix 2 presents the results of the LMM analysis showing the estimates (β) and adjusted *P* values calculated using the Benjamini–Hochberg method.

### Predicting Depression From Features

The overall performance of the ML classifiers trained with features only (ie, with no demographics data set) is listed in Table 4. Table 5 shows the ML classifier performance with the features plus age group and gender data set as predictors. The tuned hyperparameters of all ML classifiers are detailed in Multimedia Appendix 3. The performance of all 10 cross-validation folds of the features-only data set and that of the feature plus demographics data set are detailed in Multimedia Appendix 3. The performance of the DT and RWC baselines is shown in Table 6.

As shown in Table 4, it is evident that nonlinear classifiers such as XGB, RF, and KNN had superior performance in all metrics than LR. SVM (ie, SVM with RBF kernel) also performed better than LR.

In terms of precision, recall, and F1 scores, which were computed with an emphasis on the predictive performance of the positive label (ie, depressed score, PHQ-8 ≥ 10), XGB was the best performing classifier, followed by RF and KNN. XGB, RF, and KNN performed better than the RWC and DT baselines, as shown in Table 6.

Likewise, with AUC and Cohen κ performance metrics, which take into consideration both positive and negative labels (ie, nondepressed score, PHQ-8 <10), XGB, RF, and KNN classifiers had the best performance, as shown in Table 4. The AUC and Cohen κ are not biased by imbalance labels.

As shown in Table 4, the worst performing classifier is LR. Compared with the baseline classifiers in Table 6, the RWC and DT baselines classifiers outperform the LR classifier in terms of recall. The LR could predict the PHQ-8 depression score barely better than the RWC and DT baselines in all other performance metrics. The RWC baseline classifier also outperformed the SVM classifier on the recall metrics.

When age group and gender were included with features as predictors, we observed a general improvement in all performance metrics for all classifiers, as shown in Table 5. The SVM classifier had the most substantial improvement with precision increasing by 18.48% and Cohen κ increasing by 19.3%. KNN had a 6.58% improvement in precision and a 5.1% improvement in F1 score. RF and XGB classifiers had marginal improvements in all performance metrics. The worst performing classifier (ie, LR) had some gains in performance, but it could still barely outperform the DT baseline classifier in Table 6 in all performance metrics.

**Table 4:** Average and SDs of accuracy, precision, recall, F1, area under the curve, and Cohen κ metrics for 10-fold cross-validation, with features-only data set as predictors.

Metric	RF^a^, mean (SD)	XGB^b^, mean (SD)	SVM^c^, mean (SD)	LR^d^, mean (SD)	KNN^e^, mean (SD)
Accuracy	97.97 (0.37)	98.14 (0.37)	85.68 (1.16)	59.27 (1.45)	96.44 (0.52)
Precision	92.50 (1.78)	92.51 (1.25)	51.98 (2.58)	20.29 (1.25)	85.55 (1.97)
Recall	94.38 (1.86)	95.56 (1.99)	80.67 (2.36)	57.25 (4.14)	92.19 (2.24)
F1	93.41 (1.19)	94.00 (1.21)	63.20 (2.29)	29.95 (1.87)	88.73 (1.63)
Area under the curve	98.83 (0.67)	99.06 (0.54)	89.47 (1.06)	62.43 (2.22)	94.69 (1.15)
Cohen κ	92.21 (1.41)	92.90 (1.43)	54.83 (2.92)	9.66 (2.38)	86.61 (1.93)

^a^RF: random forest.

^b^XGB: XGBoost.

^c^SVM: support vector machine.

^d^LR: logistic regression.

^e^KNN: K-nearest neighbor.

**Table 5:** Average and SDs of accuracy, precision, recall, F1, area under the curve, and Cohen κ metrics for 10-fold cross-validation, with features, age group, and gender data set as predictors.

Metric	RF^a^, mean (SD)	XGB^b^, mean (SD)	SVM^c^, mean (SD)	LR^d^, mean (SD)	KNN^e^, mean (SD)
Accuracy	98.55 (0.40)	98.56 (0.31)	92.61 (0.46)	60.37 (1.39)	98.09 (0.26)
Precision	95.65 (1.59)	94.93 (1.08)	70.46 (1.63)	21.40 (1.20)	92.13 (1.41)
Recall	94.78 (1.59)	95.62 (1.52)	88.76 (3.19)	60.00 (3.94)	95.62 (1.56)
F1	95.20 (1.31)	95.27 (1.03)	78.52 (1.41)	31.54 (1.78)	93.83 (0.85)
Area under the curve	99.01 (0.51)	99.36 (0.33)	95.45 (1.00)	66.62 (3.06)	97.07 (0.73)
Cohen κ	94.34 (1.55)	94.42 (1.21)	74.13 (1.66)	11.74 (2.28)	92.69 (1.00)

^a^RF: random forest.

^b^XGB: XGBoost.

^c^SVM: support vector machine.

^d^LR: logistic regression.

^e^KNN: K-nearest neighbor.

**Table 6:** Average and SDs of accuracy, precision, recall, F1, area under the curve, and Cohen κ metrics for the RWC and DT baselines.

Metric	RWC^a^, mean (SD)	DT^b^, mean (SD)
Accuracy	25.80 (0.33)	46.80 (3.77)
Precision	15.21 (0.36)	18.70 (0.66)
Recall	84.79 (0.86)	74.33 (4.71)
F1	25.80 (0.24)	29.85 (0.75)
Area under the curve	50.00 (0.47)	62.94 (1.38)
Cohen κ	0.00 (0.32)	7.33 (1.26)

^a^RWC: random weighted classifier; RWC metrics is the average and SD of 10,000 random predictions.

^b^DT: decision tree; DT metrics is the average for 10-fold cross-validation, with age group and gender only as features.

### Feature Importance Analysis

**Figure 1:**
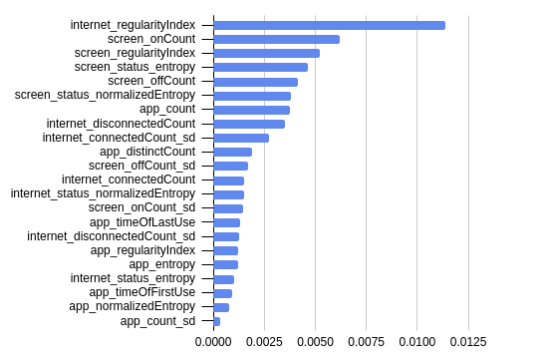
Mean permutation feature importance across 10-fold cross-validation with the XGBoost machine learning classifier.

**Figure 2:**
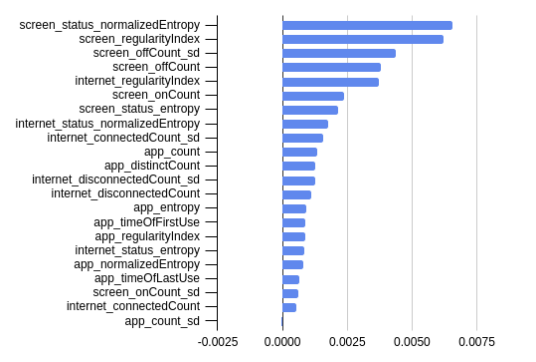
Mean permutation feature importance across 10-fold cross-validation with the random forest machine learning classifier.

**Figure 3:**
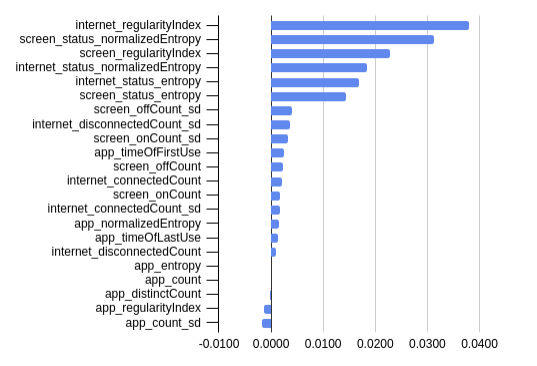
Mean permutation feature importance across 10-fold cross-validation with the K-nearest neighbor machine learning classifier.

We present the mean permutation feature importance in predicting PHQ-8 depression score across the 10-fold cross-validation with the top 3 performing ML classifiers (ie, XGB, RF, and KNN) in Figures 1-3.

For the XGB classifier in Figure 1, the top 5 most important features were the internet regularity index, screen on count, screen regularity index, screen status entropy, and the screen off count.

For the RF classifier in Figure 2, the top 5 most important features were screen status–normalized entropy, screen regularity index, screen off count SD, screen off count, and internet regularity index.

Likewise, for the KNN classifier in Figure 3, the top 5 most important features are the internet regularity index, screen status–normalized entropy, screen regularity index, internet status–normalized entropy, and the internet status entropy. As shown in Figure 3 app entropy, count, distinct count, regularity index, and count SD were less important to the KNN classifier. Removing these less important features could further improve the performance of the KNN classifier.

By ranking all important features for KNN, XGB, and RF classifiers, the top 5 most were the screen regularity index, screen status entropy, internet regularity index, screen status–normalized entropy, and the screen off count SD. App count SD is the least important feature for all classifiers (Figures 1-3), and could be removed to improve ML classifier performance in the case of RF and KNN.

## Discussion

### Overview of Data Set Employed

Our objective was to investigate the feasibility of predicting depression using multivariate digital biomarkers quantified from smartphone data sets collected in a real-world study. In this study, we used 13,898 days of smartphone data set, and 1374 PHQ-8 depression assessments from 629 participants to explore the feasibility of detecting depression from participants’ behavioral markers (ie, digital biomarkers) quantified from their smartphones. We focused on finding the relationship between repeated measures of depression scores and participant’s digital biomarkers and developing predictive models to classify depressed and nondepressed symptom severity scores.

### Principal Results

This data set was collected from a heterogeneous geographic (ie, from at least 56 different countries), occupational, and educational population, with high interindividual differences (ie, 75.84% interclass correlation).

Despite this heterogeneity, digital biomarkers extracted from participants’ smartphone data set were able to predict participants’ depression state (ie, depressed or nondepressed) with high predictive performance using ML models. The ML models achieved the following: precision, 85.55%-92.51%; recall, 92.19%-95.56%; F1, 88.73%-94.00%; AUC, 94.69%-99.06%; Cohen κ, 86.61%-92.90%; and accuracy, 96.44%-98.14%. These findings show that predictive modeling of mental health using digital biomarkers is not only possible in small homogenous populations [83,84], but also in a more general population, which further supports the scalability of this approach and its potential positive impact on health care if implemented (eg, early detection of mental disorders, RED-flag systems after treatment).

Moreover, we found that the predictive performances of ML classifiers improved when demographic characteristics were included among predictors, indicating that such variables should also be included in clinical applications. Previous studies suggest a relationship between demographic factors, smartphone usage behavior, and depression [38,85,86]. Thus, encoded in the demographic data of this study’s population is additional information that is useful in predicting the depression state of the participants. Therefore, the inclusion of additional data from clinical information systems (eg, blood parameters, previous clinical diagnosis) might be a valuable way to further increase the performance of prediction models.

Interestingly, tree-based, nearest neighbor–based classifiers had superior performance over linear classifiers, including SVM with RBF kernel, corroborating the existence of nonlinear relationships between digital biomarkers and depression. This finding further supported the correlation finding, which failed to replicate previous results reported in Saeb et al [33]. We could only identify that participants with depression symptoms were more likely to lock and unlock their phone’s screen in a random and uncertain manner (ie, significant positive correlation between screen status–normalized entropy and depression, *r*=0.14, *P*<.001). The screen status–normalized entropy biomarker quantified the frequency and distribution (ie, complexity and uncertainty) in the transition of the participants’ phone screen on and off states. All other indicators were nonsignificant. Moreover, correlation coefficients only measure the extent of the linear relationship between variables [87,88]. Instead of correlations, previous studies on mental health relied on the mutual information (MI) method from *Information* theory [87-89]. The advantage of using the MI method is that the MI measures both linear and nonlinear statistical dependencies between variables.

Given the high ICC, we tested whether LMM, a much robust method for finding linear relationships, can identify additional linear relationships. However, the results from the association analyses further showed that a unit increase in the screen status–normalized entropy positively increases the average depression score (β=.48, *P*=.03), but all other variables remained nonsignificant. This suggests that conventional statistical methods (eg, correlation or LMM) may not depict the complex nonlinear relationship between digital biomarkers and depression, and indeed more powerful methods such as ML models (eg, XGB) are needed to make better predictions [90].

The heterogeneity and inconsistency in correlation findings (ie, linear relationships) in the field are common issues [26]. Until now, it is unclear whether this is due to differences in sociodemographic characteristics in samples, in used sensors, in the method to calculate features, small sample sizes and lack of power, or even due to other between-study factors. Meta-analysis on digital markers and health outcomes (eg, depression) would be highly valuable to clearly show whether and to which extent linear relationships exist. Using meta-regression, the factors causing the differences in correlation findings may also be identified.

Nevertheless, both the correlation findings and the feature importance analysis in the prediction models clearly showed that participants’ phone screen (lock and unlock) behaviors, such as routinely and randomly locking and unlocking phone screen, and internet connectivity behaviors played the most important role in predicting their depression state. The findings in this study are also supported by prior research that investigated the relationship between screen interactions and mental health [3,27,28,33]. Passively sensed participants’ smartphone screen interaction (ie, on and off states) behavior was demonstrated to be an important predictor of mental health [27]. Similar findings have been reported previously [3,33], where the number of times a participant interacts with their phone, including screen lock and unlocks, was found to correlate with participants’ mental health state. In a neuroscience study [91], screen unlocks were found to be important behavioral markers that correlate and predict resting state brain functional connectivity, which is known to be associated with depression [92]. On internet usage behaviors, research has demonstrated an association between internet usage patterns and depression [93,94], which was also a key feature in our analysis. Thus, including these features in future studies is highly recommended.

### Limitations and Future work

Given the crowdsourced nature of the deployment of the Carat app, the sample size in the data set is small (N=629) and may not be representative of the general population. Despite the data set having a fair distribution of age groups, with a spread over several countries, it is biased toward highly educated and professional occupations. The data set is also biased toward males in gender distribution. Future research with a larger sample size and a balanced gender distribution could explore correlations, associations, and prediction performance for population subgroups.

Clinical diagnosis of depression was not an inclusion criterion for our sample population. The data set also does not contain a clinical or self-reported baseline assessment of depression and has scarce high depression scores. Because of the crowdsourced rolling recruitment nature of participants, the data set contained an unequal number of repeated depression assessments for all participants. Future research should benefit from replicating the experiment in a clinical population and a more controlled experimental design. With a clinical baseline data set, future research could study the differences in features between depressed and nondepressed groups.

The correlation and association between behavioral patterns extracted from the data set in this study and depression do not necessarily imply causal relationships. For example, the correlation between screen status–normalized entropy and depression may be caused by other confounding variables. In addition, the correlation and association between screen status–normalized entropy and depression are not strong and may not generalize in other populations. Further research is needed to establish the extent to which such behaviors cause or are a consequence of depression.

Lastly, the data set was collected from Android participants only, and the long-term use of the Carat app could influence participants’ behavior [38,39]. Research has shown that participant’s behavior and sociodemographics may differ between Android platforms and other mobile platforms such as iOS [44,95]. Future research could replicate this study to explore the extent of the differences in the participants’ behaviors (ie, digital biomarkers of participants using Android, iOS, and other mobile platforms).

Replicating the findings from this study with additional biomarkers from GPS and wearable sensor data sets and comparing their correlation and biomarker predictive importance will be interesting in future work. There would be a major design implication for depression intervention development if the behavioral markers from screen and internet connectivity achieve similar promising results as biomarkers from GPS and wearable devices. We hypothesize that screen interaction and internet connectivity data sets alone are less privacy intrusive, could better capture behaviors of immobile persons, and people may be more willing to donate such data sets to science. For example, Apple’s Screen Time and Google’s Digital Wellbeing app are processing and presenting such data to users to inform where and how users spent their time on smartphones.

### Conclusions

In summary, this study sought to find whether we can detect changes in human behavior that would be indicative of depression using smartphones. In addition, we sought to find what objective measures of human behavior from smartphones are insightful in understanding depression. Our results established a positive statistically significant linear correlation and association between depression and screen status–normalized entropy behavior quantified from smartphone data sets. Our findings also establish that behavioral markers extracted from smartphone data sets can predict whether or not a participant is depressed based on the PHQ-8 depression score, and that phone screen and internet connectivity behaviors were the most insightful behaviors that influence depression in participants. The findings in this study are supported by previous research findings and contribute to compelling evidence on the utility of digital biomarkers in augmenting traditional assessment of depression, thus enabling continuous and passive monitoring of the complex vectors of depression.

## Appendix

Multimedia Appendix 1 Description of Features Extracted from the Smartphone Dataset.

Multimedia Appendix 2 Extended Results for Statistical Analysis.

Multimedia Appendix 3 Extended Results for Machine Learning Analysis.

## Abbreviations

AUC: area under the curve
DT: decision tree
ICC: intraclass correlation
KNN: K-nearest neighbor
LMM: linear mixed model
LR: logistic regression
ML: machine learning
PHQ-8: 8-Item Patient Health Questionnaire
RF: random forest
RWC: random weighted classifier
SVM: support vector machine
XGB: XGBoost
