# Supplementary material for: Predicting Depression From Smartphone Behavioral Markers Using Machine Learning Methods, Hyperparameter Optimization, and Feature Importance Analysis: Exploratory Study
Source: JMIR Mhealth Uhealth. 2021 Jul 12;9(7):e26540. doi: 10.2196/26540 (PMC8314163; doi:10.2196/26540)
Supplement: Multimedia Appendix 1 [file mhealth_v9i7e26540_app1.pdf]

Supplementary Table 1. Description of the screen, internet connectivity and foreground application usage features extracted from participant's smartphone datasets

	Feature Name: Description
**Screen**	
	screen_offCount: count of the instances where the phone screen was off
	screen_onCount: count of instances where the phone screen was on
	screen_offCount_sd: standard deviation of the counts of instances where phone screen is off during day epochs
	screen_onCount_sd: standard deviation of the counts of instances where phone screen is on during day epochs<br>was on during day epochs
	screen_regularityIndex: regularity index of hourly screen status
	screen_status_entropy: entropy of screen status (on and off)
	screen_status_normalizedEntropy: *screen_status_entropy* divided by the log of two (ie, the number of screen states, on or off)
**Internet**	
	internet_connectedCount: count of instances where the phone was connected to the internet
	internet_disconnectedCount: count of instances where the phone was disconnected from the internet
	internet_connectedCount_sd: standard deviation of the counts of instances where the phone was connected to the internet during day epochs
	internet_disconnectedCount_sd: standard deviation of the counts of instances where the phone was disconnected from the internet during day epochs
	internet_regularityIndex: regularity index of hourly internet connectivity status
	internet_status_entropy: entropy of internet connectivity status
	internet_status_normalizedEntropy: *internet_status_entropy* divided by log two (ie, the number of internet connectivity states, connected or disconnected)
**Application**	*app_count*: count of foreground application used.
	app_distinctCount: count of distinct foreground application used

	app_timeOfFirstUse: count of minutes from hour 0 till the time the first foreground application is used.
	app_timeOfLastUse: count of minutes from hour 0 till the time the last foreground application is used.
	app_count_sd: standard deviation of the counts of foreground applications used during day epochs.
	app_entropy: entropy of the all distinct foreground application usage.
	app_normalizedEntropy: *app_entropy* divided by log of the number of distinct foreground applications used.
	app_regularityIndex: regularity index of the hourly count of distinct foreground applications used.
