# Supplementary material for: Predicting Depression From Smartphone Behavioral Markers Using Machine Learning Methods, Hyperparameter Optimization, and Feature Importance Analysis: Exploratory Study
Source: JMIR Mhealth Uhealth. 2021 Jul 12;9(7):e26540. doi: 10.2196/26540 (PMC8314163; doi:10.2196/26540)
Supplement: Multimedia Appendix 2 [file mhealth_v9i7e26540_app2.pdf]

Supplementary Table 1. Correlation between features and PHQ-8 depression score (PHQ-8) , showing Pearson's correlation coefficients, raw P values and adjusted P values using Holm-Bonferroni method

Feature	r	*P*	*P* (adjusted)
screen_status_normalizedEntropy	0.14	0.000002	0.00057
screen_status_entropy	0.09	0.001640	0.4461
app_timeOfLastUse	-0.01	0.670513	1.0000
internet_regularityIndex	-0.01	0.723636	1.0000
screen_offCount_sd	-0.01	0.754814	1.0000
internet_disconnectedCount	-0.01	0.854094	1.0000
screen_offCount	-0.01	0.859789	1.0000
app_regularityIndex	0.00	0.924839	1.0000
app_normalizedEntropy	0.00	0.902483	1.0000
internet_disconnectedCount_sd	0.01	0.840187	1.0000
app_count	0.01	0.761619	1.0000
internet_connectedCount_sd	0.01	0.696800	1.0000
app_distinctCount	0.01	0.686500	1.0000
app_entropy	0.01	0.672129	1.0000
internet_status_entropy	0.02	0.551307	1.0000
internet_status_normalizedEntropy	0.02	0.423723	1.0000
app_timeOfFirstUse	0.03	0.432404	1.0000
internet_connectedCount	0.03	0.348054	1.0000
app_count_sd	0.03	0.459091	1.0000
screen_regularityIndex	0.03	0.309170	1.0000
screen_onCount_sd	0.04	0.132285	1.0000
screen_onCount	0.06	0.046420	1.0000

Supplementary Table 2: Full correlation matrix for all features and PHQ-8 depression score, showing Pearson's correlation coefficients and adjusted P values using Holm-Bonferroni method

		app_regularityIndex	app_count	app_distinctCount	app_timeOfFirstUse	app_timeOfLastUse	app_entropy	app_normalizedEntropy	screen_offCount	screen_onCount	screen_status_entropy	screen_status_normalizedEntropy	screen_regularityIndex	internet_connectedCount	internet_disconnectedCount	internet_status_entropy	internet_status_normalizedEntropy	internet_regularityIndex	app_count_sd	internet_connectedCount_sd	internet_disconnectedCount_sd	screen_offCount_sd	screen_onCount_sd	phq8_score
**r**	app_regularityIndex	1.000	-0.280	-0.173	0.421	-0.370	-0.495	-0.269	-0.210	-0.145	-0.276	-0.095	0.138	-0.192	-0.075	-0.305	-0.063	0.345	-0.183	-0.174	-0.079	-0.169	-0.126	0.014
	app_count	-0.280	1.000	0.651	-0.236	0.229	0.544	-0.140	0.286	0.150	0.195	0.038	-0.034	0.242	0.081	0.259	0.081	-0.074	0.830	0.248	0.111	0.230	0.150	0.014
	app_distinctCount	-0.173	0.651	1.000	-0.184	0.152	0.522	-0.344	0.060	0.035	0.053	0.040	0.033	0.063	-0.031	0.088	0.107	0.005	0.612	0.040	-0.026	0.011	0.024	0.016
	app_timeOfFirstUse	0.421	-0.236	-0.184	1.000	0.071	-0.499	-0.286	-0.174	-0.134	-0.192	-0.022	0.125	-0.170	-0.044	-0.236	-0.046	0.103	-0.160	-0.156	-0.052	-0.153	-0.123	0.010
	app_timeOfLastUse	-0.370	0.229	0.152	0.071	1.000	0.359	0.201	0.097	0.083	0.188	0.086	-0.041	0.092	0.053	0.180	0.036	-0.132	0.135	0.080	0.041	0.060	0.076	-0.015
	app_entropy	-0.495	0.544	0.522	-0.499	0.359	1.000	0.300	0.261	0.178	0.253	0.034	-0.092	0.246	0.051	0.349	0.111	-0.108	0.461	0.276	0.062	0.264	0.187	0.001
	app_normalizedEntropy	-0.269	-0.140	-0.344	-0.286	0.201	0.300	1.000	0.194	0.146	0.175	-0.012	-0.081	0.173	0.115	0.224	-0.006	-0.080	-0.206	0.233	0.133	0.245	0.173	-0.028
	screen_offCount	-0.210	0.286	0.060	-0.174	0.097	0.261	0.194	1.000	0.673	0.375	-0.069	-0.101	0.918	0.291	0.578	0.066	-0.193	0.252	0.848	0.331	0.878	0.594	-0.005
	screen_onCount	-0.145	0.150	0.035	-0.134	0.083	0.178	0.146	0.673	1.000	0.641	0.343	-0.018	0.866	0.328	0.459	0.055	-0.171	0.155	0.779	0.362	0.577	0.950	0.057
	screen_status_entropy	-0.276	0.195	0.053	-0.192	0.188	0.253	0.175	0.375	0.641	1.000	0.752	-0.029	0.511	0.245	0.612	0.112	-0.288	0.173	0.542	0.246	0.380	0.659	0.090
	screen_status_normalizedEntropy	-0.095	0.038	0.040	-0.022	0.086	0.034	-0.012	-0.069	0.343	0.752	1.000	0.066	0.114	0.071	0.142	0.111	-0.130	0.034	0.101	0.074	-0.084	0.362	0.136
	screen_regularityIndex	0.138	-0.034	0.033	0.125	-0.041	-0.092	-0.081	-0.101	-0.018	-0.029	0.066	1.000	-0.073	-0.005	-0.066	0.031	0.227	-0.001	-0.080	-0.023	-0.113	-0.013	0.029
	internet_connectedCount	-0.192	0.242	0.063	-0.170	0.092	0.246	0.173	0.918	0.866	0.511	0.114	-0.073	1.000	0.130	0.631	0.184	-0.131	0.229	0.917	0.194	0.791	0.790	0.027
	internet_disconnectedCount	-0.075	0.081	-0.031	-0.044	0.053	0.051	0.115	0.291	0.328	0.245	0.071	-0.005	0.130	1.000	-0.122	-0.509	-0.358	0.054	0.102	0.904	0.292	0.332	-0.005
	internet_status_entropy	-0.305	0.259	0.088	-0.236	0.180	0.349	0.224	0.578	0.459	0.612	0.142	-0.066	0.631	-0.122	1.000	0.612	-0.057	0.235	0.712	-0.081	0.608	0.462	0.017
	internet_status_normalizedEntropy	-0.063	0.081	0.107	-0.046	0.036	0.111	-0.006	0.066	0.055	0.112	0.111	0.031	0.184	-0.509	0.612	1.000	0.353	0.076	0.225	-0.464	0.073	0.056	0.023
	internet_regularityIndex	0.345	-0.074	0.005	0.103	-0.132	-0.108	-0.080	-0.193	-0.171	-0.288	-0.130	0.227	-0.131	-0.358	-0.057	0.353	1.000	-0.046	-0.099	-0.369	-0.163	-0.145	-0.010
	app_count_sd	-0.183	0.830	0.612	-0.160	0.135	0.461	-0.206	0.252	0.155	0.173	0.034	-0.001	0.229	0.054	0.235	0.076	-0.046	1.000	0.268	0.083	0.256	0.171	0.021
	internet_connectedCount_sd	-0.174	0.248	0.040	-0.156	0.080	0.276	0.233	0.848	0.779	0.542	0.101	-0.080	0.917	0.102	0.712	0.225	-0.099	0.268	1.000	0.170	0.893	0.772	0.011
	internet_disconnectedCount_sd	-0.079	0.111	-0.026	-0.052	0.041	0.062	0.133	0.331	0.362	0.246	0.074	-0.023	0.194	0.904	-0.081	-0.464	-0.369	0.083	0.170	1.000	0.341	0.411	0.006
	screen_offCount_sd	-0.169	0.230	0.011	-0.153	0.060	0.264	0.245	0.878	0.577	0.380	-0.084	-0.113	0.791	0.292	0.608	0.073	-0.163	0.256	0.893	0.341	1.000	0.556	-0.009
	screen_onCount_sd	-0.126	0.150	0.024	-0.123	0.076	0.187	0.173	0.594	0.950	0.659	0.362	-0.013	0.790	0.332	0.462	0.056	-0.145	0.171	0.772	0.411	0.556	1.000	0.043
	phq8_score	0.014	0.014	0.016	0.010	-0.015	0.001	-0.028	-0.005	0.057	0.090	0.136	0.029	0.027	-0.005	0.017	0.023	-0.010	0.021	0.011	0.006	-0.009	0.043	1.000
P	app_regularityIndex	0.0000	0.0000	0.0000	0.0000	0.0000	0.0000	0.0000	0.0000	0.0131	0.0000	0.4988	0.0032	0.0000	1.0000	0.0000	1.0000	0.0000	1.0000	0.0000	1.0000	0.0000	0.0558	1.0000
	app_count	0.0000	0.0000	0.0000	0.0000	0.0000	0.0000	0.0053	0.0000	1.0000	0.0000	1.0000	1.0000	0.0629	1.0000	0.0000	1.0000	1.0000	0.0350	0.0009	0.0809	0.0000	1.0000	1.0000
	app_distinctCount	0.0000	0.0000	0.0000	0.0000	0.0006	0.0000	0.0000	1.0000	1.0000	1.0000	1.0000	1.0000	1.0000	1.0000	0.9723	0.1033	1.0000	0.0012	1.0000	1.0000	1.0000	1.0000	1.0000
	app_timeOfFirstUse	0.0000	0.0000	0.0000	0.0000	1.0000	0.0000	0.0000	0.0003	0.1006	0.0000	1.0000	0.0155	0.0015	1.0000	0.0000	1.0000	0.1516	1.0000	0.0012	1.0000	0.0007	0.1209	1.0000
	app_timeOfLastUse	0.0000	0.0000	0.0006	1.0000	0.0000	0.0000	0.0000	1.0000	1.0000	0.0000	1.0000	1.0000	1.0000	1.0000	0.0000	1.0000	0.0032	1.0000	1.0000	1.0000	1.0000	1.0000	1.0000
	app_entropy	0.0000	0.0000	0.0000	0.0000	0.0000	0.0000	0.0000	0.0000	0.0111	0.0000	1.0000	0.6104	0.0000	1.0000	0.0000	0.0948	0.1434	0.3849	0.0000	1.0000	0.0000	0.0014	1.0000
	app_normalizedEntropy	0.0000	0.0053	0.0000	0.0000	0.0000	0.0000	0.0000	0.0000	0.0799	0.0000	1.0000	1.0000	0.0035	0.0400	0.0000	1.0000	1.0000	0.1890	0.0000	0.0040	0.0000	0.0021	1.0000
	screen_offCount	0.0000	0.0000	1.0000	0.0003	1.0000	0.0000	0.0000	0.0000	0.0000	0.0000	1.0000	0.0864	0.0000	0.0000	0.0000	1.0000	0.0000	0.1209	0.0000	0.0000	0.0000	0.0000	1.0000
	screen_onCount	0.0131	1.0000	1.0000	0.1006	1.0000	0.0111	0.0799	0.0000	0.0000	0.0000	0.0000	1.0000	0.0000	0.0000	0.0000	1.0000	0.0000	1.0000	0.0000	0.0000	0.0000	0.0000	1.0000
	screen_status_entropy	0.0000	0.0000	1.0000	0.0000	0.0000	0.0000	0.0000	0.0000	0.0000	0.0000	0.0000	1.0000	0.0000	0.0000	0.0000	0.0191	0.0000	0.3239	0.0000	0.0000	0.0000	0.0000	0.3346
	screen_status_normalizedEntropy	0.4988	1.0000	1.0000	1.0000	1.0000	1.0000	1.0000	1.0000	0.0000	0.0000	0.0000	1.0000	0.0149	1.0000	0.0002	0.0250	0.0012	1.0000	0.0896	1.0000	0.6419	0.0000	0.0005
	screen_regularityIndex	0.0032	1.0000	1.0000	0.0155	1.0000	0.6104	1.0000	0.0864	1.0000	1.0000	1.0000	0.0000	1.0000	1.0000	1.0000	1.0000	0.0000	1.0000	0.9470	1.0000	0.0179	1.0000	1.0000
	internet_connectedCount	0.0000	0.0629	1.0000	0.0015	1.0000	0.0000	0.0035	0.0000	0.0000	0.0000	0.0149	1.0000	0.0000	0.0013	0.0000	0.0000	0.0012	1.0000	0.0000	0.0000	0.0000	0.0000	1.0000
	internet_disconnectedCount	1.0000	1.0000	1.0000	1.0000	1.0000	1.0000	0.0400	0.0000	0.0000	0.0000	1.0000	1.0000	0.0013	0.0000	0.0046	0.0000	0.0000	1.0000	0.0825	0.0000	0.0000	0.0000	1.0000
	internet_status_entropy	0.0000	0.0000	0.9723	0.0000	0.0000	0.0000	0.0000	0.0000	0.0000	0.0000	0.0002	1.0000	0.0000	0.0046	0.0000	0.0000	1.0000	0.0016	0.0000	0.8778	0.0000	0.0000	1.0000

	internet_status_normalizedEntropy	1.0000	1.0000	0.1033	1.0000	1.0000	0.0948	1.0000	1.0000	1.0000	0.0191	0.0250	1.0000	0.0000	0.0000	0.0000	0.0000	0.0000	1.0000	0.0000	0.0000	1.0000	1.0000	1.0000
	internet_regularityIndex	0.0000	1.0000	1.0000	0.1516	0.0032	0.1434	1.0000	0.0000	0.0000	0.0000	0.0012	0.0000	0.0012	0.0000	1.0000	0.0000	0.0000	1.0000	0.1108	0.0000	0.0000	0.0001	1.0000
	app_count_sd	1.0000	0.0350	0.0012	1.0000	1.0000	0.3849	0.1890	0.1209	1.0000	0.3239	1.0000	1.0000	1.0000	1.0000	0.0016	1.0000	1.0000	0.0000	0.1507	1.0000	0.0081	1.0000	1.0000
	internet_connectedCount_sd	0.0000	0.0009	1.0000	0.0012	1.0000	0.0000	0.0000	0.0000	0.0000	0.0000	0.0896	0.9470	0.0000	0.0825	0.0000	0.0000	0.1108	0.1507	0.0000	0.0000	0.0000	0.0000	1.0000
	internet_disconnectedCount_sd	1.0000	0.0809	1.0000	1.0000	1.0000	1.0000	0.0040	0.0000	0.0000	0.0000	1.0000	1.0000	0.0000	0.0000	0.8778	0.0000	0.0000	1.0000	0.0000	0.0000	0.0000	0.0000	1.0000
	screen_offCount_sd	0.0000	0.0000	1.0000	0.0007	1.0000	0.0000	0.0000	0.0000	0.0000	0.0000	0.6419	0.0179	0.0000	0.0000	0.0000	1.0000	0.0000	0.0081	0.0000	0.0000	0.0000	0.0000	1.0000
	screen_onCount_sd	0.0558	1.0000	1.0000	0.1209	1.0000	0.0014	0.0021	0.0000	0.0000	0.0000	0.0000	1.0000	0.0000	0.0000	0.0000	1.0000	0.0001	1.0000	0.0000	0.0000	0.0000	0.0000	1.0000
	phq8_score	1.0000	1.0000	1.0000	1.0000	1.0000	1.0000	1.0000	1.0000	1.0000	0.3346	0.0005	1.0000	1.0000	1.0000	1.0000	1.0000	1.0000	1.0000	1.0000	1.0000	1.0000	1.0000	0.0000

Supplementary Table 3. Results of bivariate linear mixed effect model, showing estimates (beta) and adjusted P values using Benjamini-Hochberg method

Feature	beta	*P*	*P* (adjusted)
screen_status_normalizedEntropy	0.48	0.001	0.025
screen_status_entropy	0.32	0.039	0.430
app_timeOfFirstUse	0.28	0.072	0.524
screen_regularityIndex	0.20	0.318	0.911
internet_regularityIndex	-0.20	0.326	0.911
screen_offCount	-0.12	0.342	0.911
app_entropy	-0.14	0.404	0.911
app_normalizedEntropy	-0.11	0.456	0.911
internet_disconnectedCount_sd	0.09	0.482	0.911
internet_connectedCount	-0.08	0.492	0.911
internet_disconnectedCount	0.07	0.601	0.911
internet_connectedCount_sd	-0.06	0.639	0.911
app_count_sd	0.07	0.659	0.911
screen_offCount_sd	-0.05	0.680	0.911
app_timeOfLastUse	0.05	0.689	0.911
app_distinctCount	-0.09	0.690	0.911
app_regularityIndex	0.08	0.715	0.911
app_count	-0.06	0.749	0.911
internet_status_normalizedEntropy	-0.03	0.826	0.911
internet_status_entropy	-0.03	0.828	0.911
screen_onCount_sd	0.00	0.966	0.997
screen_onCount	0.00	0.997	0.997
