# Supplementary material for: Predicting Depression From Smartphone Behavioral Markers Using Machine Learning Methods, Hyperparameter Optimization, and Feature Importance Analysis: Exploratory Study
Source: JMIR Mhealth Uhealth. 2021 Jul 12;9(7):e26540. doi: 10.2196/26540 (PMC8314163; doi:10.2196/26540)
Supplement: Multimedia Appendix 3 [file mhealth_v9i7e26540_app3.pdf]

Supplementary Table 1. Tunned Hyperparameters for Support Vector Machine, XGBoost, Random Forest, Logistic Regression and K Nearest Neighbor classifiers

Machine Learning Classifier	Hyperparameter grid values
Support Vector Machi	{<br>"C": [0.01, 0.1, 1, 10,100],<br>"gamma": ["scale","auto"],<br>"kernel": ["rbf"]<br>}
XGBoost	{<br>"learning_rate": [0.01, 0.1],<br>"n_estimators": [5, 10, 100],<br>"num_leaves": [5, 16, 31, 62]<br>}
Random Forest	{<br>'n_estimators': [2,10,100],<br>'max_depth': [3,7,9]<br>}
Logistic Regression	{<br>"C": [0.01, 0.1, 1, 10, 100],<br>"solver": ["newton-cg", "lbfgs", "liblinear", "saga"],<br>"penalty": ["l2"]<br>}
K Nearest Neighbor	{<br>"n_neighbors": [1, 3, 5],<br>"weights": ["uniform", "distance"],<br>"metric": ["euclidean", "manhattan", "minkowski"]<br>}

Supplementary Table 2. Detailed Classifier performances with features only as predictors, and hyperparameters for each fold of the cross-validation. Performance metrics for both label 1(PHQ-8 greater than or equal to 10) and label 0 are presented. The mean and standard deviation, in percentages, of all metrics are also presented. Only the mean and standard deviation metrics for Label 1 is presented in the paper

**Random Forest**	fold_id	hyperparameters	accuracy	kappa	auc	precision1	recall1	f11	precision0	recall0	f10
	1	{'max_depth': 9, 'n_estimators': 100}	0.9855	0.9435	0.9963	0.9548	0.9494	0.9521	0.9909	0.9919	0.9914
	2	{'max_depth': 9, 'n_estimators': 100}	0.9769	0.9132	0.9928	0.8953	0.9607	0.9268	0.9928	0.9798	0.9863
	3	{'max_depth': 9, 'n_estimators': 100}	0.9761	0.9055	0.9885	0.9412	0.8989	0.9195	0.9820	0.9899	0.9859
	4	{'max_depth': 9, 'n_estimators': 100}	0.9812	0.9281	0.9880	0.9239	0.9551	0.9392	0.9919	0.9859	0.9889
	5	{'max_depth': 9, 'n_estimators': 100}	0.9846	0.9412	0.9930	0.9348	0.9663	0.9503	0.9939	0.9879	0.9909
	6	{'max_depth': 9, 'n_estimators': 100}	0.9786	0.9177	0.9844	0.9227	0.9382	0.9304	0.9889	0.9859	0.9874
	7	{'max_depth': 9, 'n_estimators': 100}	0.9744	0.9024	0.9849	0.8978	0.9382	0.9176	0.9888	0.9808	0.9848
	8	{'max_depth': 9, 'n_estimators': 100}	0.9795	0.9205	0.9718	0.9326	0.9326	0.9326	0.9879	0.9879	0.9879
	9	{'max_depth': 9, 'n_estimators': 100}	0.9769	0.9116	0.9891	0.9126	0.9382	0.9252	0.9888	0.9839	0.9863
	10	{'max_depth': 9, 'n_estimators': 100}	0.9837	0.9378	0.9948	0.9344	0.9607	0.9474	0.9929	0.9879	0.9904
		Metric Mean (%)	97.97%	92.21%	98.83%	92.50%	94.38%	93.41%	98.99%	98.62%	98.80%
		Metric SD (%)	0.37%	1.41%	0.67%	1.78%	1.86%	1.19%	0.33%	0.36%	0.22%
**XGBoost**	fold_id	hyperparameters	accuracy	kappa	auc	precision1	recall1	f11	precision0	recall0	f10
	1	{'learning_rate': 0.1, 'n_estimators': 100, 'num_leaves': 5}	0.9846	0.9412	0.9958	0.9348	0.9663	0.9503	0.9939	0.9879	0.9909
	2	{'learning_rate': 0.1, 'n_estimators': 100, 'num_leaves': 5}	0.9821	0.9312	0.9921	0.9290	0.9551	0.9418	0.9919	0.9869	0.9894

	3	{'learning_rate': 0.1, 'n_estimators': 100, 'num_leaves': 5}	0.9803	0.9240	0.9847	0.9330	0.9382	0.9356	0.9889	0.9879	0.9884
	4	{'learning_rate': 0.1, 'n_estimators': 100, 'num_leaves': 5}	0.9786	0.9177	0.9823	0.9227	0.9382	0.9304	0.9889	0.9859	0.9874
	5	{'learning_rate': 0.1, 'n_estimators': 100, 'num_leaves': 5}	0.9821	0.9306	0.9891	0.9385	0.9438	0.9412	0.9899	0.9889	0.9894
	6	{'learning_rate': 0.1, 'n_estimators': 100, 'num_leaves': 5}	0.9803	0.9250	0.9879	0.9189	0.9551	0.9366	0.9919	0.9849	0.9884
	7	{'learning_rate': 0.1, 'n_estimators': 100, 'num_leaves': 5}	0.9829	0.9346	0.9887	0.9293	0.9607	0.9448	0.9929	0.9869	0.9899
	8	{'learning_rate': 0.1, 'n_estimators': 100, 'num_leaves': 5}	0.9829	0.9361	0.9967	0.9072	0.9888	0.9462	0.9980	0.9819	0.9898
	9	{'learning_rate': 0.1, 'n_estimators': 100, 'num_leaves': 5}	0.9872	0.9513	0.9994	0.9358	0.9831	0.9589	0.9969	0.9879	0.9924
	10	{'learning_rate': 0.1, 'n_estimators': 100, 'num_leaves': 5}	0.9735	0.8985	0.9892	0.9016	0.9270	0.9141	0.9868	0.9818	0.9843
		Metric Mean (%)	98.14%	92.90%	99.06%	92.51%	95.56%	94.00%	99.20%	98.61%	98.90%
		Metric SD (%)	0.37%	1.43%	0.54%	1.25%	1.99%	1.21%	0.36%	0.25%	0.22%
**Logistic Regression**	fold_id	hyperparameters	accuracy	kappa	auc	precision1	recall1	f11	precision0	recall0	f10
	1	{'C': 10, 'penalty': 'l2', 'solver': 'liblinear'}	0.6026	0.1027	0.6289	0.2065	0.5674	0.3028	0.8869	0.6089	0.7221
	2	{'C': 100, 'penalty': 'l2', 'solver': 'liblinear'}	0.5786	0.0537	0.5838	0.1805	0.5000	0.2653	0.8685	0.5927	0.7046
	3	{'C': 100, 'penalty': 'l2', 'solver': 'newton-cg'}	0.5915	0.0958	0.6424	0.2024	0.5730	0.2991	0.8859	0.5948	0.7117
	4	{'C': 100, 'penalty': 'l2', 'solver': 'lbfgs'}	0.5940	0.0734	0.6079	0.1913	0.5169	0.2792	0.8752	0.6079	0.7174
	5	{'C': 100, 'penalty': 'l2', 'solver': 'newton-cg'}	0.5966	0.1000	0.6442	0.2048	0.5730	0.3018	0.8869	0.6008	0.7163
	6	{'C': 10, 'penalty': 'l2', 'solver': 'saga'}	0.6060	0.1404	0.6531	0.2252	0.6517	0.3348	0.9053	0.5978	0.7201
	7	{'C': 100, 'penalty': 'l2', 'solver': 'newton-cg'}	0.5838	0.0966	0.6366	0.2023	0.5899	0.3013	0.8879	0.5827	0.7036
	8	{'C': 100, 'penalty': 'l2', 'solver': 'liblinear'}	0.6077	0.1119	0.5998	0.2115	0.5787	0.3098	0.8902	0.6129	0.7260
	9	{'C': 10, 'penalty': 'l2', 'solver': 'liblinear'}	0.5620	0.0797	0.6133	0.1930	0.5899	0.2909	0.8832	0.5570	0.6832
	10	{'C': 100, 'penalty': 'l2', 'solver': 'newton-cg'}	0.6048	0.1118	0.6332	0.2114	0.5843	0.3104	0.8907	0.6085	0.7230
		Metric Mean (%)	59.27%	9.66%	62.43%	20.29%	57.25%	29.95%	88.61%	59.64%	71.28%
		Metric SD (%)	1.45%	2.38%	2.22%	1.25%	4.14%	1.87%	0.97%	1.66%	1.28%
**K Nearest Neighbor**	fold_id	hyperparameters	accuracy	kappa	auc	precision1	recall1	f11	precision0	recall0	f10
	1	{'metric': 'manhattan', 'n_neighbors': 1, 'weights': 'uniform'}	0.9564	0.8337	0.9236	0.8432	0.8764	0.8595	0.9777	0.9708	0.9742
	2	{'metric': 'manhattan', 'n_neighbors': 1, 'weights': 'uniform'}	0.9590	0.8473	0.9412	0.8316	0.9157	0.8717	0.9846	0.9667	0.9756
	3	{'metric': 'manhattan', 'n_neighbors': 1, 'weights': 'uniform'}	0.9744	0.9037	0.9664	0.8854	0.9551	0.9189	0.9918	0.9778	0.9848
	4	{'metric': 'manhattan', 'n_neighbors': 1, 'weights': 'uniform'}	0.9650	0.8687	0.9494	0.8549	0.9270	0.8895	0.9867	0.9718	0.9792
	5	{'metric': 'manhattan', 'n_neighbors': 1, 'weights': 'uniform'}	0.9598	0.8515	0.9463	0.8291	0.9270	0.8753	0.9866	0.9657	0.9761
	6	{'metric': 'manhattan', 'n_neighbors': 1, 'weights': 'uniform'}	0.9658	0.8693	0.9407	0.8750	0.9045	0.8895	0.9828	0.9768	0.9798
	7	{'metric': 'manhattan', 'n_neighbors': 1, 'weights': 'uniform'}	0.9667	0.8734	0.9458	0.8717	0.9157	0.8932	0.9847	0.9758	0.9803
	8	{'metric': 'manhattan', 'n_neighbors': 1, 'weights': 'uniform'}	0.9667	0.8734	0.9458	0.8717	0.9157	0.8932	0.9847	0.9758	0.9803
	9	{'metric': 'manhattan', 'n_neighbors': 1, 'weights': 'uniform'}	0.9675	0.8796	0.9601	0.8535	0.9494	0.8989	0.9907	0.9707	0.9806
	10	{'metric': 'manhattan', 'n_neighbors': 1, 'weights': 'uniform'}	0.9624	0.8606	0.9501	0.8384	0.9326	0.8830	0.9876	0.9677	0.9776
		Metric Mean (%)	96.44%	86.61%	94.69%	85.55%	92.19%	88.73%	98.58%	97.20%	97.88%
		Metric SD (%)	0.52%	1.93%	1.15%	1.97%	2.24%	1.63%	0.40%	0.44%	0.31%
**Support Vector Machine**	fold_id	hyperparameters	accuracy	kappa	auc	precision1	recall1	f11	precision0	recall0	f10
	1	{'C': 100, 'gamma': 'scale', 'kernel': 'rbf'}	0.8470	0.5153	0.8776	0.4982	0.7697	0.6049	0.9542	0.8609	0.9051
	2	{'C': 100, 'gamma': 'scale', 'kernel': 'rbf'}	0.8598	0.5617	0.8934	0.5248	0.8315	0.6435	0.9662	0.8649	0.9128
	3	{'C': 100, 'gamma': 'scale', 'kernel': 'rbf'}	0.8684	0.5775	0.9040	0.5448	0.8202	0.6547	0.9645	0.8770	0.9187
	4	{'C': 100, 'gamma': 'scale', 'kernel': 'rbf'}	0.8444	0.5172	0.8930	0.4930	0.7921	0.6078	0.9581	0.8538	0.9030
	5	{'C': 100, 'gamma': 'scale', 'kernel': 'rbf'}	0.8769	0.5924	0.9118	0.5675	0.8034	0.6651	0.9619	0.8901	0.9246
	6	{'C': 100, 'gamma': 'scale', 'kernel': 'rbf'}	0.8385	0.5032	0.8813	0.4811	0.7865	0.5970	0.9568	0.8478	0.8990

	7	{'C': 100, 'gamma': 'scale', 'kernel': 'rbf'}	0.8530	0.5352	0.8935	0.5109	0.7921	0.6211	0.9586	0.8639	0.9088
	8	{'C': 100, 'gamma': 'scale', 'kernel': 'rbf'}	0.8650	0.5631	0.8897	0.5379	0.7978	0.6425	0.9603	0.8770	0.9168
	9	{'C': 100, 'gamma': 'scale', 'kernel': 'rbf'}	0.8580	0.5612	0.9062	0.5208	0.8427	0.6438	0.9682	0.8607	0.9113
	10	{'C': 100, 'gamma': 'scale', 'kernel': 'rbf'}	0.8571	0.5561	0.8962	0.5193	0.8315	0.6393	0.9661	0.8618	0.9109
		Metric Mean (%)	85.68%	54.83%	89.47%	51.98%	80.67%	63.20%	96.15%	86.58%	91.11%
		Metric SD (%)	1.16%	2.92%	1.06%	2.58%	2.36%	2.29%	0.46%	1.24%	0.77%

Supplementary Table 3. Detailed Classifier performances with features, age group and gender as predictors, and hyperparameters for each fold of the cross-validation. Performance metrics for both label 1(PHQ-8 greater than or equal to 10) and label 0 are presented. The mean and standard deviation, in percentages, of all metrics are also presented. Only the mean and standard deviation metrics for Label 1 is presented in the paper

**Random Forest**	fold_id	hyperparameters	accuracy	kappa	auc	precision1	recall1	f11	precision0	recall0	f10
	1	{'max_depth': 9, 'n_estimators': 100}	0.9829	0.9322	0.9826	0.9702	0.9157	0.9422	0.9850	0.9950	0.9900
	2	{'max_depth': 9, 'n_estimators': 100}	0.9786	0.9170	0.9878	0.9322	0.9270	0.9296	0.9869	0.9879	0.9874
	3	{'max_depth': 9, 'n_estimators': 100}	0.9906	0.9636	0.9960	0.9665	0.9719	0.9692	0.9950	0.9940	0.9945
	4	{'max_depth': 9, 'n_estimators': 100}	0.9872	0.9502	0.9930	0.9605	0.9551	0.9577	0.9919	0.9929	0.9924
	5	{'max_depth': 9, 'n_estimators': 100}	0.9795	0.9209	0.9838	0.9278	0.9382	0.9330	0.9889	0.9869	0.9879
	6	{'max_depth': 9, 'n_estimators': 100}	0.9863	0.9472	0.9970	0.9500	0.9607	0.9553	0.9929	0.9909	0.9919
	7	{'max_depth': 9, 'n_estimators': 100}	0.9863	0.9465	0.9870	0.9655	0.9438	0.9545	0.9900	0.9940	0.9920
	8	{'max_depth': 9, 'n_estimators': 100}	0.9872	0.9495	0.9946	0.9766	0.9382	0.9570	0.9890	0.9960	0.9925
	9	{'max_depth': 9, 'n_estimators': 100}	0.9863	0.9470	0.9876	0.9551	0.9551	0.9551	0.9919	0.9919	0.9919
	10	{'max_depth': 9, 'n_estimators': 100}	0.9897	0.9604	0.9921	0.9611	0.9719	0.9665	0.9949	0.9929	0.9939
		Metric Mean (%)	98.55%	94.34%	99.01%	95.65%	94.78%	95.20%	99.06%	99.22%	99.14%
		Metric SD (%)	0.40%	1.55%	0.51%	1.59%	1.85%	1.31%	0.33%	0.29%	0.23%
**XGBoost**	fold_id	hyperparameters	accuracy	kappa	auc	precision1	recall1	f11	precision0	recall0	f10
	1	{'learning_rate': 0.1, 'n_estimators': 100, 'num_leaves': 5}	0.9863	0.9470	0.9891	0.9551	0.9551	0.9551	0.9919	0.9919	0.9919
	2	{'learning_rate': 0.1, 'n_estimators': 100, 'num_leaves': 5}	0.9915	0.9670	0.9990	0.9667	0.9775	0.9721	0.9960	0.9940	0.9950
	3	{'learning_rate': 0.1, 'n_estimators': 100, 'num_leaves': 5}	0.9846	0.9401	0.9922	0.9545	0.9438	0.9492	0.9899	0.9919	0.9909
	4	{'learning_rate': 0.1, 'n_estimators': 100, 'num_leaves': 5}	0.9846	0.9404	0.9927	0.9494	0.9494	0.9494	0.9909	0.9909	0.9909
	5	{'learning_rate': 0.1, 'n_estimators': 100, 'num_leaves': 5}	0.9829	0.9340	0.9893	0.9389	0.9494	0.9441	0.9909	0.9889	0.9899
	6	{'learning_rate': 0.1, 'n_estimators': 100, 'num_leaves': 5}	0.9880	0.9543	0.9972	0.9457	0.9775	0.9613	0.9959	0.9899	0.9929
	7	{'learning_rate': 0.1, 'n_estimators': 100, 'num_leaves': 5}	0.9880	0.9543	0.9944	0.9457	0.9775	0.9613	0.9959	0.9899	0.9929
	8	{'learning_rate': 0.1, 'n_estimators': 100, 'num_leaves': 5}	0.9855	0.9433	0.9930	0.9600	0.9438	0.9518	0.9899	0.9929	0.9914
	9	{'learning_rate': 0.1, 'n_estimators': 100, 'num_leaves': 5}	0.9837	0.9369	0.9967	0.9492	0.9438	0.9465	0.9899	0.9909	0.9904
	10	{'learning_rate': 0.1, 'n_estimators': 100, 'num_leaves': 5}	0.9803	0.9243	0.9921	0.9282	0.9438	0.9359	0.9899	0.9869	0.9884
		Metric Mean (%)	98.56%	94.42%	99.36%	94.93%	95.62%	95.27%	99.21%	99.08%	99.15%
		Metric SD (%)	0.31%	1.21%	0.33%	1.08%	1.52%	1.03%	0.27%	0.20%	0.18%
**Logistic Regression**	fold_id	hyperparameters	accuracy	kappa	auc	precision1	recall1	f11	precision0	recall0	f10
	1	{'C': 100, 'penalty': 'l2', 'solver': 'lbfgs'}	0.6026	0.1261	0.6964	0.2181	0.6236	0.3231	0.8986	0.5988	0.7187
	2	{'C': 100, 'penalty': 'l2', 'solver': 'liblinear'}	0.5846	0.0996	0.6424	0.2038	0.5955	0.3037	0.8892	0.5827	0.7040

	3	{'C': 100, 'penalty': 'l2', 'solver': 'liblinear'}	0.6085	0.1078	0.6743	0.2095	0.5674	0.3061	0.8881	0.6159	0.7274
	4	{'C': 100, 'penalty': 'l2', 'solver': 'newton-cg'}	0.6034	0.0937	0.6218	0.2021	0.5449	0.2948	0.8826	0.6139	0.7241
	5	{'C': 100, 'penalty': 'l2', 'solver': 'newton-cg'}	0.6000	0.1352	0.6905	0.2222	0.6517	0.3314	0.9043	0.5907	0.7146
	6	{'C': 100, 'penalty': 'l2', 'solver': 'newton-cg'}	0.5786	0.0735	0.6129	0.1906	0.5449	0.2824	0.8775	0.5847	0.7018
	7	{'C': 100, 'penalty': 'l2', 'solver': 'lbfgs'}	0.6060	0.1337	0.6702	0.2220	0.6348	0.3290	0.9017	0.6008	0.7211
	8	{'C': 100, 'penalty': 'l2', 'solver': 'newton-cg'}	0.6120	0.1274	0.6764	0.2195	0.6067	0.3224	0.8968	0.6129	0.7281
	9	{'C': 100, 'penalty': 'l2', 'solver': 'newton-cg'}	0.6262	0.1308	0.6764	0.2227	0.5843	0.3225	0.8946	0.6337	0.7419
	10	{'C': 100, 'penalty': 'l2', 'solver': 'newton-cg'}	0.6151	0.1463	0.7012	0.2291	0.6461	0.3382	0.9055	0.6095	0.7286
		Metric Mean (%)	60.37%	11.74%	66.62%	21.40%	60.00%	31.54%	89.39%	60.44%	72.10%
		Standard Deviation (%)	1.39%	2.28%	3.06%	1.20%	3.94%	1.78%	0.94%	1.59%	1.20%
**K Nearest Neighbor**	fold_id	hyperparameters	accuracy	kappa	auc	precision1	recall1	f11	precision0	recall0	f10
	1	{'metric': 'manhattan', 'n_neighbors': 1, 'weights': 'uniform'}	0.9786	0.9189	0.9690	0.9091	0.9551	0.9315	0.9919	0.9829	0.9873
	2	{'metric': 'manhattan', 'n_neighbors': 1, 'weights': 'uniform'}	0.9821	0.9315	0.9733	0.9243	0.9607	0.9421	0.9929	0.9859	0.9894
	3	{'metric': 'manhattan', 'n_neighbors': 1, 'weights': 'uniform'}	0.9778	0.9146	0.9615	0.9176	0.9382	0.9278	0.9889	0.9849	0.9869
	4	{'metric': 'manhattan', 'n_neighbors': 1, 'weights': 'uniform'}	0.9846	0.9409	0.9748	0.9396	0.9607	0.9500	0.9929	0.9889	0.9909
	5	{'metric': 'manhattan', 'n_neighbors': 1, 'weights': 'uniform'}	0.9795	0.9223	0.9718	0.9096	0.9607	0.9344	0.9929	0.9829	0.9878
	6	{'metric': 'manhattan', 'n_neighbors': 1, 'weights': 'uniform'}	0.9786	0.9174	0.9597	0.9274	0.9326	0.9300	0.9879	0.9869	0.9874
	7	{'metric': 'manhattan', 'n_neighbors': 1, 'weights': 'uniform'}	0.9855	0.9448	0.9822	0.9305	0.9775	0.9534	0.9959	0.9869	0.9914
	8	{'metric': 'manhattan', 'n_neighbors': 1, 'weights': 'uniform'}	0.9803	0.9263	0.9792	0.9016	0.9775	0.9380	0.9959	0.9808	0.9883
	9	{'metric': 'manhattan', 'n_neighbors': 1, 'weights': 'uniform'}	0.9820	0.9303	0.9641	0.9435	0.9382	0.9408	0.9889	0.9899	0.9894
	10	{'metric': 'manhattan', 'n_neighbors': 1, 'weights': 'uniform'}	0.9795	0.9223	0.9718	0.9096	0.9607	0.9344	0.9929	0.9828	0.9878
		Metric Mean (%)	98.09%	92.69%	97.07%	92.13%	95.62%	93.83%	99.21%	98.53%	98.87%
		Metric SD (%)	0.26%	1.00%	0.73%	1.41%	1.56%	0.85%	0.28%	0.29%	0.16%
**Support Vector Machine**	fold_id	hyperparameters	accuracy	kappa	auc	precision1	recall1	f11	precision0	recall0	f10
	1	{'C': 100, 'gamma': 'scale', 'kernel': 'rbf'}	0.9179	0.7172	0.9515	0.6767	0.8820	0.7659	0.9776	0.9244	0.9503
	2	{'C': 100, 'gamma': 'scale', 'kernel': 'rbf'}	0.9214	0.7161	0.9496	0.7048	0.8315	0.7629	0.9688	0.9375	0.9529
	3	{'C': 100, 'gamma': 'scale', 'kernel': 'rbf'}	0.9291	0.7411	0.9497	0.7317	0.8427	0.7833	0.9710	0.9446	0.9576
	4	{'C': 100, 'gamma': 'scale', 'kernel': 'rbf'}	0.9256	0.7389	0.9423	0.7040	0.8820	0.7830	0.9778	0.9335	0.9551
	5	{'C': 100, 'gamma': 'scale', 'kernel': 'rbf'}	0.9256	0.7452	0.9580	0.6936	0.9157	0.7893	0.9840	0.9274	0.9549
	6	{'C': 100, 'gamma': 'scale', 'kernel': 'rbf'}	0.9342	0.7679	0.9600	0.7285	0.9045	0.8070	0.9821	0.9395	0.9603
	7	{'C': 100, 'gamma': 'scale', 'kernel': 'rbf'}	0.9274	0.7480	0.9612	0.7031	0.9045	0.7912	0.9819	0.9315	0.9560
	8	{'C': 100, 'gamma': 'scale', 'kernel': 'rbf'}	0.9256	0.7463	0.9650	0.6920	0.9213	0.7904	0.9850	0.9264	0.9548
	9	{'C': 100, 'gamma': 'scale', 'kernel': 'rbf'}	0.9239	0.7317	0.9388	0.7014	0.8708	0.7769	0.9757	0.9334	0.9541
	10	{'C': 100, 'gamma': 'scale', 'kernel': 'rbf'}	0.9307	0.7608	0.9704	0.7100	0.9213	0.8020	0.9851	0.9324	0.9580
		Metric Mean (%)	92.61%	74.13%	95.47%	70.46%	88.76%	78.52%	97.89%	93.31%	95.54%
		Metric SD (%)	0.46%	1.66%	1.00%	1.63%	3.19%	1.41%	0.58%	0.62%	0.28%
